# Impact of Serum Retinol-binding Protein 4 Levels in Late Pregnancy on the Incidence of Small/Large for Gestational Age Infants Among 11,854 Pregnant Women: A Retrospective Study

**DOI:** 10.2188/jea.JE20240275

**Published:** 2025-06-05

**Authors:** Bin Zhang, Zhaolong Zhan, Sijie Xi, Feng Wang, Xiaosong Yuan

**Affiliations:** 1 Department of Medical Genetics, Changzhou Maternal and Child Health Care Hospital, Changzhou Medical Center, Nanjing Medical University, Changzhou, China; 2 Department of Obstetrics and Gynecology, Yancheng Third People’s Hospital, Yancheng, China

**Keywords:** retinol-binding protein 4, birth weight, small for gestational age, large for gestational age, prediction

## Abstract

**Background:**

There is still uncertainty regarding the association between maternal serum levels of retinol-binding protein 4 (RBP4) and pregnancy outcomes. This study aimed to determine the association between RBP4 levels and incidence of small/large for gestational age (SGA/LGA) infants among the Chinese population.

**Methods:**

This was a retrospective study of 11,854 pregnant women who delivered at Changzhou Maternal and Child Health Care Hospital between 2016 and 2017 and whose serum RBP4 levels were measured at the time of admission. The incidence of SGA/LGA deliveries was retrieved from the medical records of the participants.

**Results:**

Maternal RBP4 levels in the second, third, and fourth quartiles (28.8–34.0, 34.1–40.0, and >40.0 mg/L, respectively) were associated with lower birthweights relative to those in the first quartile (<28.8 mg/L), with estimated average decreases of 51.30 g (95% confidence interval [CI], −70.51 to −32.10), 86.86 g (95% CI, −106.50 to −67.22) and 124.08 g (95% CI, −144.51 to −103.64), respectively (*P* for trend <0.01). Pregnant women in the fourth quartile for RBP4 levels had a greater SGA risk (odds ratio [OR] 2.14; 95% CI, 1.72–2.65) and lower LGA risk (OR 0.53; 95% CI, 0.45–0.63) than those in the first quartile after controlling for demographic variables, gestational age, pregnancy complications, and other laboratory results. The sensitivity analysis indicated the consistency of these findings.

**Conclusion:**

High RBP4 levels in late pregnancy are associated with an increased SGA risk and decreased LGA risk, indicating that serum RBP4 levels at the time of admission for delivery could be a promising predictor of SGA/LGA delivery.

## INTRODUCTION

There is growing evidence suggesting a close relationship between birthweight and adult susceptibility to metabolic dysfunction and cardiovascular disease.^1^^,^^2^ Birthweight according to gestational week in newborns is assessed on the basis of growth reference percentiles, and neonates are categorized as large for gestational age (LGA), appropriate for gestational age (AGA), or small for gestational age (SGA).^3^ Several reports have focused on the health outcomes of LGA and SGA newborns, revealing a similar increased risk of diabetes, cardiovascular disease, and cancer.^4^^–^^6^ Most notably, many of these abnormalities have been observed in individuals who are SGA or LGA not only during childhood and adolescence but also during adulthood, suggesting that alterations in fetal birthweight may have important implications for health outcomes. Therefore, it is crucial to predict and prevent the occurrence of SGA and LGA.

Retinol-binding protein 4 (RBP4), also known as a vitamin A transport polypeptide, is a soluble 21 kDa protein that is synthesized primarily in white adipose tissue and the liver. In the circulation, it functions as a main blood carrier of retinol, a crucial molecule in DNA transcription, embryonic development and fetal growth, representing the potential to strengthen hepatic gluconeogenesis and decrease insulin sensitivity.^7^ Elevated circulating RBP4 levels have been observed in several metabolic and endocrine syndromes, such as cardiovascular disease, obesity, type 2 diabetes mellitus (T2DM), and polycystic ovary syndrome.^8^^,^^9^ Although RBP4 is frequently measured in clinical practice, its normal reference interval for specific populations (especially for pregnant women) remains unclear. A number of studies investigating maternal plasma or serum RBP4 levels during pathological pregnancies, such as gestational diabetes mellitus (GDM), preeclampsia (PE), and SGA or LGA neonates, have yielded inconsistent results. For example, some studies have reported increased serum RBP4 levels in GDM and PE patients, while others have indicated decreased levels or no significant differences/associations.^10^^–^^15^ In addition, several studies with small sample sizes (ranging from 29 to 480 participants) investigated the association between RBP4 levels during pregnancy and subsequent risk of delivery of SGA/LGA neonates, and a positive association (RBP4 and SGA) and a negative association (RBP4 and LGA) were observed in some studies but not in others.^15^^–^^19^ To date, no epidemiological cohort studies have reported potential RBP4-associated alterations in patients with intrahepatic cholestasis of pregnancy (ICP).

The aim of this retrospective study was to describe the distribution of maternal serum RBP4 levels at admission for hospital delivery and to investigate the association of RBP4 levels with SGA/LGA risk, exploring whether maternal serum RBP4 levels could be treated as a potential predictive marker of SGA/LGA.

## METHODS

### Study design and participants

The study participants were from a single-center cohort of 13,275 consecutive pregnant women who delivered at Changzhou Maternal and Child Health Care Hospital during one recruitment period from April 1, 2016 to March 31, 2017. The women admitted to our hospital for delivery were enrolled in this cohort, and the birth outcomes and health of the mother-offspring pairs were recorded at delivery and 7 days postpartum from their medical records. This study included participants from the retrospective study population, and the exclusion criteria were as follows: 1) multiple gestation; 2) history of main pregestational diseases, such as diabetes, hypertension, heart, liver, kidney, thyroid, autoimmune disorder, and syphilis; 3) stillbirth and fetal malformations; 4) consumption of illicit drugs, tobacco, and alcohol during pregnancy; and 5) missing RBP4 levels at admission for hospital delivery. None of the participants used alcohol or smoked during pregnancy in this study. Among the 13,275 participants who were enrolled, 1,691 who presented with multiple gestations (*n* = 335), a history of pregestational diseases (*n* = 488), fetal death or malformations (*n* = 96), or without RBP4 levels (*n* = 752) were excluded. Ultimately, 11,854 participants were eligible for the data analysis.

This study was performed in accordance with the principles of the Declaration of Helsinki. Approval was granted by the Ethics Committee of Changzhou Maternal and Child Health Care Hospital (ZD201803). The need to obtain participants’ written informed consent was waived due to the retrospective nature of the study and the analysis of anonymous data from our electronic medical records system. The reporting of the current retrospective study followed the Strengthening the Reporting of Observational Studies in Epidemiology guidelines.^20^

### Data collection and laboratory analysis

The medical information on maternal demographics, gravidity, parity, pregestational medical history (eg, hypertension, diabetes), delivery mode, neonatal gestational age, sex, birth length and birthweight, and admission/discharge diagnosis were retrieved from the medical records. The laboratory findings were collected from the laboratory information system of our hospital. Serum hepatic and renal function, lipid profiles, and high-sensitivity C-reactive protein (hsCRP) levels were measured at the time of admission to hospital delivery for each participant. Although RBP4 levels have been routinely measured in clinical practice in recent years, the normal reference intervals for pregnant women remain unclear. Under the sponsorship of the reagent’s manufacturer, RBP4 levels were routinely investigated in the same sample. The biochemical parameters were determined using automatic analyzers with matched reagents (for hepatic and renal function, lipid profiles and RBP4: AU5800, Beckman Coulter Inc., Tokyo, Japan; for hsCRP: BN II System, Siemens Diagnostics Inc., Erlangen, Germany).

### Diagnosis and definitions

GDM, ICP, PE, and pregnancy-induced hypertension (PIH) were regarded as the main pregnancy complications, while preterm birth (PTB), SGA, and LGA were considered to be major adverse birth outcomes. The pregnancy complications in the study population were diagnosed according to previous reports.^21^^,^^22^ PTB was defined as delivery before 37 gestational weeks.^23^ Based on the gestational age and birthweight, neonates were stratified into three groups: 1) SGA, defined as a birthweight <10^th^ percentile for a specific week of gestational age based on a previous report; 2) AGA, defined as birthweight in the 10–90^th^ percentile; and 3) LGA, defined as birthweight >90^th^ percentile.^3^ According to previous studies, potential confounding variables that might affect the association between maternal RBP4 levels during late pregnancy and SGA/LGA delivery were selected as follows: maternal age (≤20, 20–34 and ≥35 years), prenatal body mass index (BMI) (<25, 25–29 and ≥30 kg/m^2^), parity (multipara and primipara), fertilization method (natural fertility and assisted reproduction technology use), and pregnancy complications [GDM, ICP, PE, PIH, and nonpregnancy complications (NPC)].^24^^–^^26^

### Statistical analysis

Continuous variables are reported as the mean and standard deviation (SD), while categorical variables are shown as the frequency and percentage. The demographic characteristics and laboratory findings of the participants in this study are described in quartiles of maternal serum RBP4 levels. Comparisons among different RBP4 groups (quartiles) were performed using analysis of variance/Kruskal-Wallis tests for continuous variables and chi-squared tests for categorical variables. The distributions of the serum RBP4 levels during late pregnancy according to the pregnancy outcomes were determined, and the medians were compared using a nonparametric method.^27^ Spearman rank analysis was applied to assess the correlation of RBP4 levels with maternal characteristics and laboratory findings. The associations between RBP4 levels and fetal growth indices (birth length, birthweight, and gestational age) and between RBP4 levels and SGA/LGA were determined as categorical variables using quartiles, with the bottom quartile as the reference group. General linear and logistic regression models were used to calculate the regression coefficient (β), odds ratio (OR) and 95% confidence interval (CI). The multivariate models were as follows: model 1, unadjusted; model 2, adjusted for maternal age, BMI, parity, systolic and diastolic BP, pregnancy complications, gestational age, assisted reproduction technology use, and fetal sex; and model 3, adjusted for model 2 variables plus laboratory findings. Subgroup analysis was performed to explore whether the association between RBP4 levels and SGA/LGA varied by age, BMI, parity, assisted reproduction technology use, and pregnancy complications. The curves of receiver operating characteristic (ROC) were depicted to analyze the area under the curve (AUC) obtained from RBP4, other laboratory indicators and predict models in detecting the delivery of infants with SGA and LGA.

All the data were analyzed with Empower software version 2.0 (X&Y Solutions, Inc., Boston, MA, USA) and the R statistical package (R Foundation for Statistical Computing, Vienna, Austria). A *P* value <0.05 was considered to indicate statistical significance.

## RESULTS

### Participants’ characteristics

A total of 11,854 mother-and-singleton-newborn pairs were eligible for the final data analysis. Approximately 8.4% (999/11,854), 6.2% (733/11,854), 3.6% (426/11,854), and 2.1% (251/11,854) of the women were diagnosed with GDM, ICP, PE, and PIH, respectively. Of the 11,854 singleton newborns, 1,053 (8.9%) and 1,843 (15.5%) were classified as SGA and LGA, respectively. The mean maternal age and prenatal BMI at delivery were 28.6 (SD, 4.4) years and 27.3 (SD, 3.4) kg/m^2^, respectively. A total of 7,110 (60.0%) pregnant women were nulliparous. The newborns in this retrospective study had a mean gestational age of 38.7 (SD, 1.7) weeks, a mean birth length of 49.8 (SD, 1.4) cm, and a mean birthweight of 3,339.3 (SD, 498.5) g. Table 1 presents the demographic characteristics and laboratory findings of the analyzed women by quartiles of serum RBP4 levels. The ranges of serum RBP4 levels for quartiles (Q) 1–4 was <28.8, 28.8–34.0, 34.1–40.0, and >40.0 mg/L, respectively. There were significant differences in maternal age, BMI, parity, blood pressure, gestational age at delivery, assisted reproduction technology use, pregnancy complications (except for PIH), fetal sex, birth length and birthweight, and laboratory findings among the different RBP4 groups. In addition, the incidence of SGA births exhibited a significant incremental trend from Q1 to Q4, while that of LGA births exhibited the opposite trend (SGA: from 6.4% to 11.7%; LGA: from 20.0% to 12.8%).

**Table 1.** Characteristics of study population and laboratory tests by quartiles of maternal serum RBP4 levels (*N* = 11,854)

	RBP4 (mg/L)				*P* value

	Q1 (<28.8, *N* = 2,951)	Q2 (28.8–34.0, *N* = 2,953)	Q3 (34.1–40, *N* = 2,960)	Q4 (>40, *N* = 2,990)	
Maternal characteristics					
Age, years, mean (SD)	28.3 (4.4)	28.5 (4.4)	28.8 (4.5)	28.9 (4.5)	<0.001
<20	33 (1.1%)	38 (1.3%)	22 (0.7%)	20 (0.7%)	0.028
20–34	2,695 (91.3%)	2,682 (90.8%)	2,689 (90.8%)	2,694 (90.1%)	
≥35	223 (7.6%)	233 (7.9%)	249 (8.4%)	276 (9.2%)	
BMI, kg/m^2^,^a^ mean (SD)	26.9 (3.3)	27.2 (3.4)	27.5 (3.4)	27.7 (3.4)	<0.001
<25	862 (29.6%)	781 (26.8%)	678 (23.1%)	607 (20.4%)	<0.001
25–29	1,562 (53.7%)	1,582 (54.3%)	1,627 (55.4%)	1,658 (55.8%)	
≥30	484 (16.6%)	553 (19.0%)	632 (21.5%)	704 (23.7%)	
Parity					
No child	1,825 (61.8%)	1,795 (60.8%)	1,737 (58.7%)	1,753 (58.6%)	0.024
≥1 child	1,126 (38.2%)	1,158 (39.2%)	1,223 (41.3%)	1,237 (41.4%)	
Systolic BP, mm Hg, mean (SD)	120.4 (11.6)	120.8 (12.2)	120.9 (12.0)	121.8 (12.7)	0.001
Diastolic BP, mm Hg, mean (SD)	74.2 (8.1)	74.5 (8.3)	74.5 (8.3)	75.0 (8.7)	0.017
Gestational age, weeks, mean (SD)	38.5 (1.9)	38.7 (1.7)	38.8 (1.5)	38.8 (1.6)	<0.001
Assisted reproduction	59 (2.0%)	64 (2.2%)	59 (2.0%)	97 (3.2%)	0.003
Delivery mode					
Vaginal delivery	1,734 (58.8%)	1,731 (58.6%)	1,690 (57.1%)	1,623 (54.3%)	0.001
Cesarean section	1,217 (41.2%)	1,222 (41.4%)	1,270 (42.9%)	1,367 (45.7%)	
Pregnancy complications^b^					
GDM	280 (9.5%)	254 (8.6%)	250 (8.4%)	215 (7.2%)	0.016
ICP	238 (8.1%)	195 (6.6%)	151 (5.1%)	149 (5.0%)	<0.001
PE	93 (3.2%)	108 (3.7%)	95 (3.2%)	130 (4.3%)	0.049
PIH	70 (2.4%)	53 (1.8%)	56 (1.9%)	72 (2.4%)	0.2
PTB	312 (10.6%)	202 (6.8%)	173 (5.8%)	160 (5.4%)	<0.001
Newborn characteristics					
Sex					
Female	1,284 (43.5%)	1,353 (45.8%)	1,417 (47.9%)	1,542 (51.6%)	<0.001
Male	1,667 (56.5%)	1,600 (54.2%)	1,543 (52.1%)	1,448 (48.4%)	
Birth length, cm, mean (SD)	49.7 (1.6)	49.8 (1.4)	49.9 (1.2)	49.8 (1.5)	0.011
Birth weight, g, mean (SD)	3,364.5 (538.6)	3,347.6 (494.1)	3,339.6 (466)	3,306.0 (491.2)	<0.001
Weight for gestational age					
SGA	190 (6.4%)	240 (8.1%)	272 (9.2%)	351 (11.7%)	<0.001
AGA	2,172 (73.6%)	2,266 (76.7%)	2,263 (76.5%)	2,257 (75.5%)	
LGA	589 (20.0%)	447 (15.1%)	425 (14.4%)	382 (12.8%)	
Laboratory tests					
Total bilirubin, µmol/L, mean (SD)	8.1 (3.1)	7.9 (3.0)	7.8 (2.9)	7.8 (3.0)	<0.001
Direct bilirubin, µmol/L, mean (SD)	1.7 (1.2)	1.6 (1.0)	1.5 (0.9)	1.5 (1.0)	<0.001
ALT, U/L, mean (SD)	11.5 (16.4)	11.0 (12.9)	11.1 (9.4)	12.7 (15.4)	<0.001
AST, U/L, mean (SD)	20.4 (13.0)	19.8 (11.1)	19.6 (8.0)	20.9 (25.5)	<0.001
Total protein, g/L, mean (SD)	61.9 (4.2)	63.0 (4.2)	64.0 (4.2)	65.0 (4.4)	<0.001
Albumin, g/L, mean (SD)	35.8 (2.6)	36.3 (2.4)	36.6 (2.4)	37.0 (2.5)	0.009
Urea nitrogen, mmol/L, mean (SD)	3.4 (0.9)	3.5 (0.9)	3.6 (0.9)	3.7 (1.0)	<0.001
Creatinine, umol/L, mean (SD)	59.6 (9.9)	59.6 (8.4)	60.1 (8.1)	61.2 (9.6)	<0.001
Total cholesterol, mmol/L, mean (SD)	6.1 (1.2)	6.4 (1.2)	6.4 (1.2)	6.6 (1.3)	<0.001
Triglyceride, mmol/L, mean (SD)	3.5 (1.5)	3.8 (1.8)	4.0 (1.8)	4.4 (2.0)	<0.001
LDL-C, mmol/L, mean (SD)	3.3 (0.9)	3.4 (0.9)	3.4 (0.9)	3.4 (1.0)	<0.001
HDL-C, mmol/L, mean (SD)	1.6 (0.3)	1.7 (0.3)	1.8 (0.3)	1.8 (0.4)	<0.001
hsCRP, mg/L, mean (SD)	5.6 (9.7)	4.0 (4.6)	3.9 (3.8)	3.9 (3.6)	<0.001

ALT, alanine aminotransferase; AST, aspartate aminotransferase; BMI, body mass index; BP, blood pressure; GDM, gestational diabetes mellitus; HDL-C, high density lipoprotein cholesterol; hsCRP, high sensitive C-reactive protein; ICP, intrahepatic cholestasis of pregnancy; LDL-C, low density lipoprotein cholesterol; N, number; PE, pre-eclampsia; PIH, pregnancy induced hypertension; PTB, preterm birth; Q, quartile; RBP4, retinol-binding protein 4; SGA/AGA/LGA, small/appropriate/large for gestational age; SD, standard deviation.

^a^124 women did not calculate BMI due to height or weigh loss.

^b^234 participants were diagnosed with two types of pregnancy complications.

### Comparison of maternal serum RBP4 levels based on pregnancy outcomes

The distributions of the 11,854 maternal serum RBP4 levels as a function of pregnancy complications, birth outcomes, and across different gestational weeks are shown in Table 2 and eTable 1, with values for the 5^th^, 10^th^, 25^th^, 50^th^, 75^th^, 90^th^, and 95^th^ percentiles. Serum RBP4 levels were significantly different between the GDM/ICP/PE group and the NPC group (median: 33.1/31.8/34.9 vs 34.2 mg/L, all *P* < 0.05). The median RBP4 level was significantly lower in the PTB group than in the full-term birth (FTB) group (31.3 vs 34.3 mg/L, *P* < 0.01). Additionally, compared with that in the AGA group, a higher RBP4 value was observed in the SGA group, whereas a lower RBP4 value was found in the LGA group (median: 36.1/32.6 vs 34.2 mg/L, all *P* < 0.01).

**Table 2.** The distribution of maternal serum RBP4 levels on admission for labor based on pregnancy outcomes

	*N*	P 5	P 10	P 25	Median	P 75	P 90	P 95	*P* value
All women^a^	11,854	22.3	24.6	28.8	34.1	40.1	46.2	50.3	
NPC	9,679	22.5	24.8	28.9	34.2	40.2	46.2	50.2	
GDM	999	21.8	23.8	28.1	33.1	39.2	45.6	49.8	<0.001^b^
ICP	733	20.7	22.8	27.3	31.8	38.3	45.8	50.4	<0.001^b^
PE	426	20.8	23.4	29.4	34.9	42.6	47.8	55.3	0.047^b^
PIH	251	22.1	23.8	28.3	34.8	41.3	47.2	52.2	0.3^b^
FTB	11,007	22.5	24.8	29.0	34.3	40.2	46.3	50.4	
PTB	847	19.1	21.7	26.4	31.3	38.0	44.6	48.3	<0.001^c^
AGA	8,958	22.4	24.8	28.9	34.2	40.1	46.0	50.1	
SGA	1,053	23.4	26.3	30.1	36.1	42.5	49.2	55.0	<0.001^d^
LGA	1,843	21.3	23.1	27.2	32.6	38.9	44.7	48.3	<0.001^d^

FTB, full term birth; GDM, gestational diabetes mellitus; ICP, intrahepatic cholestasis of pregnancy; NPC, non-pregnancy complications; P, percentile; PE, pre-eclampsia; PIH, pregnancy induced hypertension; PTB, pre-term birth; RBP4, retinol-binding protein 4; SGA/AGA/LGA, small/appropriate/large for gestational age.

^a^234 participants were diagnosed with two types of pregnancy complications.

^b^Compared with NPC group.

^c^Compared with FTB group.

^d^Compared with AGA group.

### Maternal serum RBP4 levels, demographics, and results of the routine biochemical analysis

According to the correlation analysis, RBP4 levels were significantly positively correlated with maternal age, BMI, parity, blood pressure, gestational age, alanine aminotransferase levels, aspartate aminotransferase levels, total protein levels, urea nitrogen levels, creatinine levels, and blood lipid levels during late pregnancy. Additionally, correlation analysis revealed significant negative correlations of RBP4 levels with total bilirubin, direct bilirubin, and hsCRP levels (Table 3).

**Table 3.** Correlation of RBP4 with maternal characteristics and laboratory results at hospitalization for delivery

	*r*	*P* value
Age	0.055	<0.001
BMI	0.094	<0.001
Systolic BP	0.034	<0.001
Diastolic BP	0.032	<0.001
Parity	0.032	<0.001
Gestational age	0.056	<0.001
Total bilirubin, µmol/L	−0.051	<0.001
Direct bilirubin, µmol/L	−0.120	<0.001
ALT, U/L	0.142	<0.001
AST, U/L	0.032	<0.001
Total Protein, g/L	0.278	<0.001
Albumin, g/L	0.187	0.1
Urea nitrogen, mmol/L	0.141	<0.001
Creatinine, umol/L	0.081	<0.001
Total cholesterol, mmol/L	0.143	<0.001
Triglyceride, mmol/L	0.220	<0.001
LDL-C, mmol/L	0.039	<0.001
HDL-C, mmol/L	0.168	<0.001
hsCRP, mg/L	−0.024	0.011

ALT, alanine aminotransferase; AST, aspartate aminotransferase; BMI, body mass index; BP, blood pressure; HDL-C, high density lipoprotein cholesterol; hsCRP, high sensitive C-reactive protein; LDL-C, low density lipoprotein cholesterol; RBP4, retinol-binding protein 4.

### Maternal serum RBP4 levels and fetal growth, SGA, and LGA

The associations between RBP4 levels and indicators of fetal growth are described in Table 4. Compared with women in the lowest quartile of RBP4 levels, women in the highest quartile of RBP4 levels had a lower birth length. The βs were 0.05 (95% CI, −0.03 to 0.12) in the unadjusted model (model 1). However, the association became significant in model 2 after adjusting for maternal age, BMI, parity, blood pressure, pregnancy complications, use of assisted reproduction technology, and fetal sex (β, −0.13; 95% CI, −0.18 to −0.07), and in model 3, which was further adjusted for laboratory findings (β, 0.04; 95% CI, −0.04 to 0.13). Maternal RBP4 levels in Q2, Q3, and Q4 were associated with lower birthweight relative to that in Q1, with estimated mean decreases of 51.30 g (95% CI, −70.51 to −32.10), and 86.86 g (95% CI, −106.50 to −67.22; *P* < 0.01), and 124.08 g (95% CI, −144.51 to −103.64), respectively (all *P* values for trend <0.01). The associations of SGA/LGA risk with RBP4 levels classified into quartiles among pregnant women are also presented in Table 4. According to the crude models (model 1), an increased risk of SGA and a decreased risk of LGA were found in pregnant women with RBP4 levels in Q4 (>40.0 mg/L) compared with those in Q1 (<28.8 mg/L), with ORs of 1.93 (95% CI, 1.61–2.32) and 0.59 (95% CI, 0.51–0.68), respectively. In models 2 and 3, after controlling for potential confounding factors, the RBP4–SGA/LGA association was slightly enhanced, with ORs of 2.19 (95% CI, 1.81–2.66) and 0.51 (95% CI, 0.44–0.59), and ORs of 2.14 (95% CI, 1.72–2.65) and 0.53 (95% CI, 0.45–0.63), respectively. These associations remained consistent in the sensitivity analysis among participants who were not of advanced age (eTable 2), who were not obese (eTable 3), who were primipara (eTable 4), who had not used assisted reproduction technology (eTable 5), who had NPC (eTable 6), and who had FTB (eTable 7).

**Table 4.** Associations of serum RBP4 levels with fetal growth indicators and SGA/LGA

	Birth length (cm)		Birthweight (g)		SGA		LGA	

	β (95% CI)	*P* value	β (95% CI)	*P* value	OR (95% CI)	*P* value	OR (95% CI)	*P* value
Model 1^a^								
Q1 (<28.8 mg/L)	Ref.		Ref.		Ref.		Ref.	
Q2 (28.8–34.0 mg/L)	0.06 (−0.01 to 0.13)	0.1	−16.96 (−42.38 to 8.45)	0.2	1.29 (1.06–1.57)	0.013	0.72 (0.62–0.82)	<0.001
Q3 (34.1–40.1 mg/L)	0.12 (0.05–0.20)	0.001	−24.95 (−50.35 to 0.45)	0.054	1.47 (1.21–1.78)	<0.001	0.67 (0.59–0.77)	<0.001
Q4 (>40.1 mg/L)	0.05 (−0.03 to 0.12)	0.2	−58.57 (−83.90 to −33.23)	<0.001	1.93 (1.61–2.32)	<0.001	0.59 (0.51–0.68)	<0.001
*P* for trend		0.2		<0.001		<0.001		<0.001
Continuous	0.00 (0.00–0.01)	0.1	−2.62 (−3.64 to −1.60)	<0.001	1.03 (1.02–1.03)	<0.001	0.98 (0.97–0.98)	<0.001
Model 2^b^								
Q1 (<28.8 mg/L)	Ref.		Ref.		Ref.		Ref.	
Q2 (28.8–34.0 mg/L)	−0.03 (−0.09 to 0.02)	0.2	−52.41 (−71.57 to −33.24)	<0.001	1.33 (1.08–1.63)	0.007	0.68 (0.59–0.79)	<0.001
Q3 (34.1–40.1 mg/L)	−0.06 (−0.11 to −0.00)	0.042	−94.35 (−113.56 to −75.15)	<0.001	1.65 (1.35–2.02)	<0.001	0.60 (0.52–0.69)	<0.001
Q4 (>40.1 mg/L)	−0.13 (−0.18 to −0.07)	<0.001	−131.49 (−150.73 to −112.25)	<0.001	2.19 (1.81–2.66)	<0.001	0.51 (0.44–0.59)	<0.001
*P* for trend		<0.001		<0.001		<0.001		<0.001
Continuous	−0.01 (−0.01 to 0.00)	<0.001	−5.85 (−6.62 to −5.08)	<0.001	1.03 (1.03–1.04)	<0.001	0.97 (0.96–0.98)	<0.001
Model 3^c^								
Q1 (<28.8 mg/L)	Ref.		Ref.		Ref.		Ref.	
Q2 (28.8–34.0 mg/L)	−0.05 (−0.11 to 0.01)	0.072	−51.30 (−70.51 to −32.10)	<0.001	1.33 (1.07–1.65)	0.009	0.69 (0.60–0.81)	<0.001
Q3 (34.1–40.1 mg/L)	−0.07 (−0.13 to −0.01)	0.013	−86.86 (−106.50 to −67.22)	<0.001	1.60 (1.29–1.99)	<0.001	0.64 (0.55–0.75)	<0.001
Q4 (>40.1 mg/L)	−0.15 (−0.21 to −0.09)	<0.001	−124.08 (−144.51 to −103.64)	<0.001	2.14 (1.72–2.65)	<0.001	0.53 (0.45–0.63)	<0.001
*P* for trend		<0.001		<0.001		<0.001		<0.001
Continuous	−0.01 (−0.01 to −0.00)	<0.001	−5.74 (−6.58 to −4.91)	<0.001	1.03 (1.02–1.04)	<0.001	0.97 (0.97–0.98)	<0.001

BMI–body mass index; BP–blood pressure; CI–confidence interval; hsCRP–high sensitive C-reactive protein; OR–odds ratio; Q–quartile; RBP4–retinol-binding protein 4; SGA/LGA–small/large for gestational age.

^a^Unadjusted.

^b^Adjusted for maternal age–BMI–systolic and diastolic BP–parity–gestational week–pregnancy complications–assisted reproduction and fetal sex.

^c^Adjusted for model 2 variables plus blood lipids–hsCRP–liver and kidney function.

### Stratified analysis by potential covariates

To further demonstrate that the findings in Table 4 are robust to potential confounding factors, stratified analyses were performed by subgroups defined by the main covariates known to potentially affect SGA/LGA risk, including age, BMI, parity, pregnancy complications, and assisted reproduction technology use. All the statistical analyses were adjusted for age, BMI, gestational week, parity, blood pressure, pregnancy complications, assisted reproduction technology use, fetal sex, and laboratory findings, except for the stratified variate. As shown in Table 5 and Table 6, the associations between increased RBP4 levels and SGA/LGA risk were consistent in most subgroups. Interestingly, the highest risk of SGA and the lowest risk of LGA were observed among pregnant women with PE and an RBP4 level >40.0 mg/L, with ORs of 9.54 (95% CI, 5.76–15.81) and 0.25 (95% CI, 0.12–0.52), respectively, compared with those among NPC women with RBP4 levels ≤40.0 mg/L.

**Table 5.** Subgroup analysis of effective modifications of clinical characteristics on associations between RBP4 and SGA infants

	Low RBP4 (Q1–Q3)		High RBP4 (Q4)		Crude		Adjusted^a^	

	Total	SGA (%)	Total	SGA (%)	OR (95% CI)	*P* value	OR (95% CI)	*P* value
Age, years								
<35	7,865	638 (8.1%)	2,611	316 (12.1%)	1.56 (1.35–1.80)	<0.001	1.62 (1.37–1.90)	<0.001
≥35	999	64 (6.4%)	379	35 (9.2%)	1.15 (0.81–1.65)	0.4	1.31 (0.87–1.98)	0.2
BMI, kg/m^2 b^								
<30	7,092	631 (8.9%)	2,265	295 (13.0%)	1.53 (1.32–1.78)	<0.001	1.39 (1.18–1.64)	<0.001
≥30	1,669	58 (3.5%)	704	51 (7.2%)	0.80 (0.59–1.08)	0.1	0.65 (0.47–0.90)	0.009
Parity								
No child	5,357	493 (9.2%)	1,753	226 (12.9%)	1.46 (1.23–1.73)	<0.001	1.55 (1.28–1.87)	<0.001
≥1 child	3,507	209 (6.0%)	1,237	125 (10.1%)	1.11 (0.90–1.36)	0.3	1.31 (1.02–1.68)	0.031
Assisted reproduction								
No	8,682	694 (8.0%)	2,893	345 (12.0%)	1.56 (1.36–1.79)	<0.001	1.58 (1.35–1.85)	<0.001
Yes	182	8 (4.4%)	97	6 (6.2%)	0.76 (0.33–1.74)	0.5	1.05 (0.44–2.49)	0.9
Pregnancy complications^c^								
No	7,210	561 (7.8%)	2,469	264 (10.7%)	1.42 (1.22–1.66)	<0.001	1.48 (1.24–1.76)	<0.001
GDM	784	39 (5.0%)	215	17 (7.9%)	1.02 (0.62–1.68)	0.9	1.23 (0.72–2.12)	0.5
ICP	584	58 (9.9%)	149	22 (14.8%)	2.05 (1.30–3.25)	0.002	1.80 (1.07–3.03)	0.026
PE	296	58 (19.6%)	130	47 (36.2%)	6.71 (4.64–9.70)	<0.001	9.54 (5.76–15.81)	<0.001
PIH	179	13 (7.3%)	72	10 (13.9%)	1.91 (0.97–3.75)	0.059	2.77 (1.32–5.84)	0.007

BMI, body mass index; BP, blood pressure; CI, confidence interval; GDM, gestational diabetes mellitus; ICP, intrahepatic cholestasis of pregnancy; OR, odds ratio; PE, pre-eclampsia; PIH, pregnancy induced hypertension; Q, quartile; RBP4, retinol-binding protein 4; SGA, small for gestational age.

^a^Adjusted for maternal age, BMI, parity, gestational week, systolic and diastolic BP, pregnancy complications, assisted reproduction, fetal sex, blood lipids, hsCRP, liver and kidney function, except for the variable that was categorized.

^b^BMI was not provided in 124 women due to missing height or weight.

^c^234 pregnant women were diagnosed with two complications.

**Table 6.** Subgroup analysis of effective modifications of clinical characteristics on associations between RBP4 and LGA infants

	Low RBP4 (Q1–Q3)		High RBP4 (Q4)		Crude		Adjusted^a^	

	Total	LGA (%)	Total	LGA (%)	OR (95% CI)	*P* value	OR (95% CI)	*P* value
Age, years								
<35	7,865	1,199 (15.2%)	2,611	306 (11.7%)	0.74 (0.65–0.84)	<0.001	0.71 (0.61–0.82)	<0.001
≥35	999	262 (26.2%)	379	76 (20.1%)	1.39 (1.08–1.81)	0.012	0.93 (0.69–1.26)	0.7
BMI, kg/m^2 b^								
<30	7,092	955 (13.5%)	2,265	212 (9.4%)	0.66 (0.57–0.78)	<0.001	0.74 (0.63–0.88)	0.001
≥30	1,669	495 (29.7%)	704	165 (23.4%)	1.97 (1.63–2.37)	<0.001	2.00 (1.63–2.46)	<0.001
Parity								
No child	5,357	672 (12.5%)	1,753	178 (10.2%)	0.79 (0.66–0.94)	0.008	0.78 (0.65–0.94)	0.011
≥1 child	3,507	789 (22.5%)	1,237	204 (16.5%)	1.38 (1.16–1.63)	<0.001	1.01 (0.83–1.24)	0.9
Assisted reproduction								
No	8,682	1,420 (16.4%)	2,893	361 (12.5%)	0.73 (0.64–0.83)	<0.001	0.71 (0.62–0.82)	<0.001
Yes	182	41 (22.5%)	97	21 (21.7%)	1.41 (0.87–2.30)	0.2	1.10 (0.64–1.89)	0.7
Pregnancy complications^c^								
No	7,210	1,073 (14.9%)	2,469	307 (12.4%)	0.81 (0.71–0.93)	0.003	0.79 (0.68–0.92)	0.003
GDM	784	238 (30.4%)	215	45 (20.9%)	1.51 (1.08–2.12)	0.016	1.12 (0.77–1.64)	0.6
ICP	584	103 (17.6%)	149	12 (8.1%)	0.50 (0.28–0.91)	0.022	0.44 (0.23–0.85)	0.014
PE	296	56 (18.9%)	130	12 (9.2%)	0.58 (0.32–1.06)	0.075	0.25 (0.12–0.52)	<0.001
PIH	179	34 (19.0%)	72	16 (22.2%)	1.63 (0.93–2.86)	0.085	1.30 (0.70–2.42)	0.4

BMI, body mass index; BP, blood pressure; CI, confidence interval; GDM, gestational diabetes mellitus; ICP, intrahepatic cholestasis of pregnancy; LGA, large for gestational age; OR, odds ratio; PE, pre-eclampsia; PIH, pregnancy induced hypertension; Q, quartile; RBP4, retinol-binding protein 4.

^a^Adjusted for maternal age, BMI, parity, gestational week, systolic and diastolic BP, pregnancy complications, assisted reproduction, fetal sex, blood lipids, hsCRP, liver and kidney function, except for the variable that was categorized.

^b^BMI was not provided in 124 women due to missing height or weight.

^c^234 pregnant women were diagnosed with two complications.

### Ability to predict SGA and LGA using RBP4 and related models

**Figure 1.**
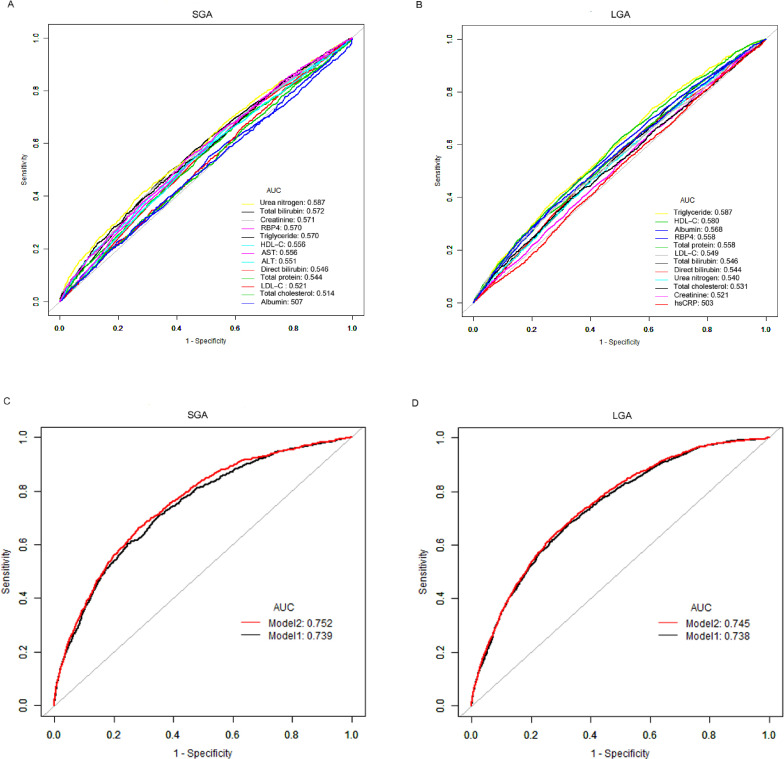
ROC curves analysis to compare RBP4, laboratory indices, and models to predict SGA/LGA.

ROC analyses were performed to determine the best thresholds of RBP4, related laboratory indicators and models for detecting infants with SGA and LGA (Figure 1). The optimal cutoffs of RBP4 for detecting the delivery of SGA and LGA infants were 35.5 and 29.7 mg/L, with the areas under the AUC of 0.570 and 0.558 (Table 7). In addition, we investigated further into two models that incorporate clinical factors and laboratory measurements for predicting SGA and LGA deliveries. Model 1 included maternal age, BMI, BP, parity, gestational age, pregnancy complications, assisted reproduction, and the results of laboratory measurements except for RBP4. By incorporating RBP4 into model 1 (resulting in model 2), the AUC for predicting the delivery of SGA infants significantly improved from 0.739 to 0.752 (*P* < 0.001), and similarly, for LGA infants, the AUC improved from 0.738 to 0.745 (*P* < 0.001) (Figure 1 and Table 7).

**Table 7.** Accuracy of RBP4, laboratory indices, and models to predict SGA/LGA

	AUC	95% CI	*P* value^a^	Best threshold	Specificity (%)	Sensitivity (%)	PPV (%)	NPV (%)
SGA								
Urea nitrogen, mmol/L	0.587	0.569–0.606	0.2	3.9	71.6	41.5	12.5	92.6
Total bilirubin, µmol/L	0.572	0.554–0.591	0.9	6.7	62.8	49.1	11.4	92.7
Creatinine, umol/L	0.571	0.553–0.589	1.0	62.5	66.1	45.0	11.5	92.5
RBP4, mg/L	0.570	0.552–0.589		35.5	57.1	53.9	10.9	92.7
Triglyceride, mmol/L	0.570	0.551–0.588	1.0	3.0	70.9	39.6	11.7	92.3
HDL-C, mmol/L	0.556	0.537–0.575	0.3	1.9	70.2	38.8	11.3	92.2
AST, U/L	0.556	0.538–0.574	0.3	18.3	53.7	55.0	10.4	92.4
ALT, U/L	0.551	0.553–0.569	0.1	8.5	42.6	66.4	10.2	92.9
Direct bilirubin, µmol/L	0.546	0.528–0.564	0.049	1.7	37.5	70.3	9.9	92.8
Total protein, g/L	0.544	0.526–0.563	0.026	64.8	63.4	43.8	10.5	92.0
LDL-C, mmol/L	0.521	0.503–0.539	<0.001	3.0	37.7	66.3	9.5	91.9
Total cholesterol, mmol/L	0.514	0.495–0.532	<0.001	7.6	84.4	18.7	10.5	91.4
Albumin, g/L	0.507	0.488–0.526	<0.001	36.3	49.2	54.8	9.5	91.8
Model 1^b^	0.739	0.723–0.755		−2.4	64.6	71.1	16.3	95.8
Model 2^c^	0.752	0.736–0.767	<0.001	−2.2	71.5	66.4	18.4	95.7
LGA								
Triglyceride, mmol/L	0.587	0.573–0.601	0.007	3.2	38.8	73.8	18.1	89.0
HDL-C, mmol/L	0.580	0.566–0.595	0.010	1.7	49.6	62.5	18.5	87.8
Albumin, g/L	0.568	0.553–0.582	0.4	35.6	64.7	46.1	19.4	86.7
RBP4, mg/L	0.558	0.544–0.573		29.7	72.6	36.6	19.7	86.2
Total protein, g/L	0.558	0.544–0.572	1.0	60.7	75.4	32.8	19.7	85.9
LDL-C, mmol/L	0.549	0.535–0.564	0.6	2.9	70.2	38.2	19.0	86.1
Total bilirubin, µmol/L	0.546	0.532–0.560	0.3	6.3	32.1	75.6	17.1	87.7
Direct bilirubin, µmol/L	0.544	0.530–0.559	0.2	1.6	59.2	48.9	18.1	86.3
Urea nitrogen, mmol/L	0.540	0.526–0.554	0.056	3.3	59.5	46.7	17.5	85.9
Total cholesterol, mmol/L	0.531	0.516–0.546	0.011	5.9	65.4	41.0	17.8	85.8
Creatinine, umol/L	0.521	0.507–0.535	<0.001	61.7	39.4	64.6	16.4	85.8
hsCRP, mg/L	0.503	0.489–0.518	<0.001	3.2	47.0	54.8	15.9	85.0
Model 1^b^	0.738	0.726–0.751		−1.7	67.1	68.2	27.6	92.0
Model 2^c^	0.745	0.733–0.757	<0.001	−1.6	71.3	64.9	29.4	91.7

ALT, alanine aminotransferase; AST, aspartate aminotransferase; AUC, area under the curve; CI, confidence interval; HDL-C, high density lipoprotein cholesterol; hsCRP, high sensitive C-reactive protein; LDL-C, low density lipoprotein cholesterol; NPV, negative predictive value; PPV, positive predictive value; RBP4, retinol-binding protein 4; SGA/LGA, small/large for gestational age.

^a^*P* values indicated the significance of differences between RBP4 and laboratory indices or between Model 1 and Model 2.

^b^Model 1 included age, BMI, systolic and diastolic BP, parity, gestational week, pregnancy complications, assisted reproduction, and laboratory indices.

^c^Model 2, model 1 plus RBP4.

## DISCUSSION

To the best of our knowledge, this is the largest retrospective study to assess the association between RBP4 levels and the incidence of SGA/LGA delivery. In this hospital-based observational study, we comprehensively plotted the maternal serum RBP4 level distribution by pregnancy outcomes in Chinese women and found a significant negative association between the maternal serum RBP4 level and fetal birth length and weight and a significantly decreased risk of LGA and an increased risk of SGA elicited by high RBP4 levels (>40.1 mg/L). Subgroup analyses among the participants with nonadvanced age, primipara, natural fertility, and no pregnancy complications revealed consistent results. Additionally, compared with that in NPC women with an RBP4 level <40.1 mg/L, the incidence of SGA increased from 7.8% to 35.7% in PE patients with high RBP4 levels, representing an absolute risk increase of 27.9% and a relative risk increase of 7.1-fold. These findings demonstrate that the serum RBP4 level in late pregnancy could be a promising indicator of the risk of SGA/LGA.

During normal pregnancy, Ueland observed a significant increase in plasma RBP4 levels from early (14–16 weeks) to late pregnancy (30–32 weeks) in 44 Norwegian normal pregnant women, whereas Chan et al and Inoue et al did not find such an increase related to gestational stage in 20 Chinese and 16 Japanese healthy pregnant women, respectively.^28^^–^^30^ In this large retrospective study, the maternal serum RBP4 level was positively influenced by maternal characteristics at 28–41 weeks of gestation, including age, BMI, BP, parity and gestational age, which was not in accordance with a previous study conducted in the United Kingdom, in which no correlation of the RBP4 level with maternal BMI and gestational age was found in 240 normal women at 11 to 13 weeks of gestation.^18^ However, Chan et al observed a positive correlation between the serum RBP4 level and BMI.^29^ In addition, the plasma RBP4 level was demonstrated to be positively and significantly correlated with BMI and BP in nonpregnant Chinese adults.^31^^,^^32^ Previous studies in pathological pregnancies in patients with PE and GDM have reported that the maternal plasma/serum RBP4 level could be elevated, reduced, and not significantly different from that of normal control patients.^10^^–^^15^ In this observational study, increased serum RBP4 levels were observed in PE pregnancies, and RBP4 levels were positively correlated with indices of hepatic and renal function. These findings support the hypothesis that the liver dysfunction (increased synthesis) and kidney dysfunction (less excretion) that occur in PE are likely to be the main source of increased RBP4 levels.^33^^,^^34^ GDM also complicates pregnancies and shares a similar pathogenesis with T2DM. RBP4 levels were anticipated to be elevated in pregnant women with GDM. In contrast, this study found that pregnant women diagnosed with GDM had decreased RBP4 levels, which has been observed in two previous studies from Austria and Germany.^35^^,^^36^ In addition, other studies reported no significant change in RBP4 levels between GDM patients and controls.^37^^,^^38^

Several studies with small sample sizes (cases <500) have investigated the association between fetal birthweight and RBP4 levels in cord blood and reported inconsistent results, with five studies suggesting a positive association and one report indicating no association.^29^^,^^30^^,^^39^^–^^42^ Notably, two studies showed that plasma RBP4 levels during late pregnancy or serum RBP4 levels at 11–13 weeks of gestation were higher or not changed in pregnant women who subsequently delivered SGA infants.^18^^,^^19^ Another three studies demonstrated that pregnant women who delivered SGA neonates had decreased serum RBP4 levels in the first trimester of pregnancy and those who delivered LGA infants had increased plasma RBP4 levels in the third trimester.^15^^–^^17^ In contrast, the present study suggested that, among 11,854 Chinese pregnant women (9,679 healthy and 2,175 with pregnancy complications), serum RBP4 levels in the top quartile (compared with the bottom quartile) before hospital labor were significantly correlated with deceased birth length and weight, with an increased risk of delivering an SGA neonate and a reduced risk of delivering an LGA neonate. These contradictory findings concerning RBP4 levels in maternal plasma/serum or cord blood in both pathological and normal pregnancies could be attributed to the difference among the studies in terms of the study population, epidemiological design, sample size, timing of sample collection, specimen type, assay technique, and control of confounding factors. For example, the association between RBP4 gene single nucleotide polymorphisms and GDM risk is inconsistent among different ethnic populations.^43^^,^^44^ Additionally, different detection methods (eg, ELISA, Western blot, liquid chromatography–mass spectrometry, and immunoturbidimetry) contribute to laboratory variations in RBP4 levels.^45^ Longitudinal prospective cohort studies to investigate alterations in RBP4 levels across the whole pregnancy and its association with pregnancy outcomes are urgently needed.

Our study has several important strengths that are worth mentioning. First, this observational study provides crucial evidence of significant associations between RBP4 levels and birth length and weight. Second, this study further evaluated the association between high RBP levels and an increased risk of SGA in a large sample of more than 11,000 pregnant Chinese women. Third, this study was the first to show that pregnant women with ICP had decreased serum RBP4 levels before labor. Finally, the participants’ medical records were relatively complete, the diagnosis of pregnancy outcomes and obstetric care were implemented by the same professional team, and all the tests were performed in the same laboratory, which made the results reliable. In addition, detailed potential confounding factors, including maternal demographic variables, reproductive history, pregnancy complications, and liver and kidney function indicators, blood lipids, and hsCRP, were included in the relevant adjustments.

One inevitable limitation of this observational study is its retrospective design. The reason why the participants in this study were administered RBP4 serum tests was the establishment of normal reference intervals (rather than well-designed criteria). We attempted to reduce selection bias by excluding major causes (chronic hepatic and renal disease) likely to influence RBP4 levels and birthweight, but the bias may still be present. The other limitation is that detailed data on family socioeconomic status, physical exercise habits, dietary habits, prepregnancy BMI, and gestational weight gain are absent from our database, which could bias the associations observed in this research. Therefore, further prospective and longitudinal cohort studies with well-designed protocols are necessary to confirm and extrapolate our findings and to investigate whether RBP4 level-modifying therapies are able to prevent birthweight-related disorders.

### Conclusion

This largest real-world analysis comprehensively demonstrated the maternal serum RBP4 distribution by pregnancy outcomes and revealed that elevated serum RBP4 levels in late pregnancy are associated with an increased risk of SGA delivery and a decreased risk of LGA delivery, and the RBP4-associated SGA/LGA risk could be modified by pregnancy complications. These findings suggest that serum RBP4 measurement at the time of hospitalization for labor might be a promising predictive marker for SGA/LGA delivery, representing a more scientific foundation to prevent these birthweight-altered disorders, which might be beneficial in an obstetric management and clinical care.

## SUPPLEMENTARY MATERIAL

eTable 1. The distribution of maternal serum RBP4 levels on admission for labor based on gestational weeks

eTable 2. Associations of serum RBP4 levels with fetal growth indicators and SGA/LGA among participants without advance age (N = 10,476)

eTable 3. Associations of serum RBP4 levels with fetal growth indicators and SGA/LGA among participants without obesity (N = 9,357)

eTable 4. Associations of serum RBP4 levels with fetal growth indicators and SGA/LGA among nulliparous participants (N = 7,110)

eTable 5. Associations of serum RBP4 levels with fetal growth indicators and SGA/LGA among participants with natural fertility (N = 11,575)

eTable 6. Associations of serum RBP4 levels with fetal growth indicators and SGA/LGA among participants without pregnancy complications (N = 9,679)

eTable 7. Associations of serum RBP4 levels with fetal growth indicators and SGA/LGA among participants without preterm birth (N = 11,007)
