# Supplementary material for: Impact of Serum Retinol-binding Protein 4 Levels in Late Pregnancy on the Incidence of Small/Large for Gestational Age Infants Among 11,854 Pregnant Women: A Retrospective Study
Source: J Epidemiol. 2025 Jun 5;35(6):287–96. doi: 10.2188/jea.JE20240275 (PMC12066191; doi:10.2188/jea.JE20240275)
Supplement: Supplementary file 1 [file je-35-287-s001.pdf]

eTable 1. The distribution of maternal serum RBP4 levels on admission for labor based on gestational weeks

Gestational week	N	P 5	P 10	P 25	Median	P 75	P 90	P 95
28	9	16.4	19.3	22.8	25.6	37.4	47.1	47.8
29	22	18.1	18.6	26.4	29.7	38.5	47.5	49.9
30	25	25.4	27.3	28.5	35.1	44.6	53.9	57.0
31	39	18.8	19.3	25.8	28.8	39.0	46.0	47.7
32	63	15.0	20.5	28.0	30.9	35.5	42.2	44.0
33	78	17.6	20.8	25.8	30.4	36.3	44.7	48.5
34	120	21.2	23.2	27.4	32.3	38.0	43.8	47.8
35	178	19.4	21.8	25.3	30.9	37.3	44.6	47.7
36	313	20.4	21.8	26.4	32.5	38.5	44.0	47.7
37	734	21.3	23.7	28.1	33.2	39.2	45.6	50.5
38	2,886	22.4	24.7	28.8	34.2	40.4	46.2	50.4
39	3,847	22.9	25.0	29.2	34.4	40.1	46.4	50.4
40	2,528	22.7	25.0	29.1	34.3	40.4	46.5	50.0
41	1,012	22.2	24.8	28.9	34.7	40.6	46.4	50.2

P, percentile; RBP4, retinol-binding protein 4.

eTable 2. Associations of serum RBP4 levels with fetal growth indicators and SGA/LGA among participants without advance age (N=10,476)

	Birth length, cm		Birthweight, g		SGA		LGA	
	β (95% CI)	*P* value	β (95% CI)	*P* value	OR (95% CI)	*P* value	OR (95% CI)	*P* value
Model 1[a]								
Q1 (<28.8 mg/L)	Ref.		Ref.		Ref.		Ref.	
Q2 (28.8–34.0 mg/L)	0.05 (-0.03 to 0.12)	0.2	-17.91 (-44.61 to 8.79)	0.2	1.21 (1.05–1.58)	0.017	0.70 (0.60–0.81)	<0.001
Q3 (34.1–40.1 mg/L)	0.12 (0.04–0.20)	0.003	-23.19 (-50.01 to 3.63)	0.090	1.46 (1.19–1.79)	<0.001	0.66 (0.57–0.77)	<0.001
Q4 (>40.1 mg/L)	0.03 (-0.04 to 0.11)	0.4	-55.34 (-82.10 to -28.58)	<0.001	1.94 (1.60–2.36)	<0.001	0.58 (0.49–0.67)	<0.001
P for trend				<0.001		<0.001		<0.001
Continuous	0.00 (-0.00 to 0.00)	0.3	-2.59 (-3.66 to -1.51)	<0.001	1.03 (1.02–1.04)	<0.001	0.97 (0.97–0.98)	<0.001
Model 2[b]								
Q1 (<28.8 mg/L)	Ref.		Ref.		Ref.		Ref.	
Q2 (28.8–34.0 mg/L)	-0.05 (-0.11 to 0.01)	0.1	-51.65 (-71.73 to -31.56)	<0.001	1.31 (1.05–1.62)	0.015	0.68 (0.58–0.79)	<0.001
Q3 (34.1–40.1 mg/L)	-0.06 (-0.12 to -0.01)	0.031	-92.79 (-113.01 to -72.56)	<0.001	1.62 (1.31–2.00)	<0.001	0.60 (0.51–0.71)	<0.001
Q4 (>40.1 mg/L)	-0.13 (-0.19 to -0.08)	<0.001	-125.90 (-146.15 to -105.65)	<0.001	2.19 (1.79–2.69)	<0.001	0.51 (0.43–0.60)	<0.001
P for trend		<0.001		<0.001		<0.001		<0.001
Continuous	-0.01 (-0.01 to 0.00)	<0.001	-5.79 (-6.60 to -4.97)	<0.001	1.03 (1.03–1.04)	<0.001	0.97 (0.96–0.98)	<0.001
Model 3[c]								
Q1 (<28.8 mg/L)	Ref.		Ref.		Ref.		Ref.	

Q2 (28.8–34.0 mg/L)	-0.06 (-0.12 to -0.00)	0.037	-51.53 (-71.64 to -31.41)	<0.001	1.31 (1.05–1.64)	0.019	0.69 (0.58–0.81)	<0.001
Q3 (34.1–40.1 mg/L)	-0.08 (-0.14 to -0.02)	0.014	-87.87 (-108.55 to -67.20)	<0.001	1.60 (1.28–2.01)	<0.001	0.63 (0.53–0.74)	<0.001
Q4 (>40.1 mg/L)	-0.15 (-0.21 to -0.09)	<0.001	-121.96 (-143.44 to -100.49)	<0.001	2.17 (1.74–2.72)	<0.001	0.52 (0.43–0.62)	<0.001
P for trend		<0.001		<0.001		<0.001		<0.001
Continuous	-0.01 (-0.01 to 0.00)	<0.001	-5.87 (-6.75 to -4.99)	<0.001	1.04 (1.03–1.05)	<0.001	0.97 (0.96–0.98)	<0.001

BMI, body mass index; BP, blood pressure; CI, confidence interval; hsCRP, high sensitive C-reactive protein; OR, odds ratio; Q, quartile; RBP4, retinol-binding protein 4; SGA/LGA, small/large for gestational age.

[a] Unadjusted.

[b] Adjusted for maternal BMI, parity, systolic and diastolic BP, gestational week, pregnancy complications, assisted reproduction and fetal sex.

[c] Adjusted for model 2 variables plus blood lipids, hsCRP, liver and kidney function.

eTable 3. Associations of serum RBP4 levels with fetal growth indicators and SGA/LGA among participants without obesity (N=9,357)

	Birth length, cm [d]		Birthweight, g [d]		SGA [d]		LGA [d]	
	β (95% CI)	*P* value	β (95% CI)	*P* value	OR (95% CI)	*P* value	OR (95% CI)	*P* value
Model 1[a]								
Q1 (<28.8 mg/L)	Ref.		Ref.		Ref.		Ref.	
Q2 (28.8–34.0 mg/L)	0.11 (0.03–0.18)	0.009	-0.34 (-27.23–26.55)	1.0	1.24 (1.01–1.53)	0.044	0.74 (0.63–0.88)	<0.001
Q3 (34.1–40.1 mg/L)	0.14 (0.06–0.22)	0.001	-24.79 (-51.85–2.27)	0.073	1.57 (1.28–1.93)	<0.001	0.65 (0.55–0.77)	<0.001
Q4 (>40.1 mg/L)	0.06 (-0.02 to 0.14)	0.1	-64.59 (-91.78 to -37.41)	<0.001	1.94 (1.59–2.36)	<0.001	0.53 (0.44–0.63)	<0.001
P for trend		0.1		<0.001		<0.001		<0.001
Continuous	0.00 (-0.00 to 0.01)	0.1	-3.14 (-4.23 to -2.04)	<0.001	1.03 (1.02–1.04)	<0.001	0.97 (0.96–0.98)	<0.001
Model 2[b]								
Q1 (<28.8 mg/L)	Ref.		Ref.		Ref.		Ref.	
Q2 (28.8–34.0 mg/L)	-0.01 (-0.07 to 0.05)	0.8	-37.04 (-58.13 to -15.94	0.001	1.24 (1.00–1.54)	0.046	0.75 (0.64–0.89)	0.001
Q3 (34.1–40.1 mg/L)	-0.03 (-0.09 to 0.03)	0.3	-77.78 (-99.08 to -56.49)	<0.001	1.59 (1.30–1.96)	<0.001	0.65 (0.55–0.78)	<0.001
Q4 (>40.1 mg/L)	-0.10 (-0.16 to -0.04)	0.001	-115.44 (-136.90 to -93.98)	<0.001	1.90 (1.55–2.33)	<0.001	0.54 (0.45–0.64)	<0.001
P for trend		0.001		<0.001		<0.001		<0.001
Continuous	-0.00 (-0.01 to -0.00)	<0.001	-5.32 (-6.19 to -4.46)	<0.001	1.03 (1.02–1.04)	<0.001	0.97 (0.95–0.99)	<0.001
Model 3[c]								
Q1 (<28.8 mg/L)	Ref.		Ref.		Ref.		Ref.	
Q2 (28.8–34.0 mg/L)	-0.02 (-0.08 to 0.04)	0.5	-32.64 (-53.77 to -11.50)	0.003	1.22 (0.98–1.52)	0.082	0.78 (0.66–0.93)	0.006

Q3 (34.1–40.1 mg/L)	-0.05 (-0.11 to 0.02)	0.2	-67.99 (-89.72 to -46.26)	<0.001	1.52 (1.22–1.90)	<0.001	0.71 (0.59–0.85)	<0.001
Q4 (>40.1 mg/L)	-0.12 (-0.19 to -0.05)	<0.001	-102.80 (-125.55 to -80.05)	<0.001	1.79 (1.43–2.24)	<0.001	0.58 (0.48–0.71)	<0.001
P for trend		<0.001		<0.001		<0.001		<0.001
Continuous	-0.01 (-0.01 to -0.00)	0.005	-5.10 (-6.03 to -4.16)	<0.001	1.03 (1.02–1.04)	<0.001	0.98 (0.97–0.98)	<0.001

BMI, body mass index; BP, blood pressure; CI, confidence interval; hsCRP, high sensitive C-reactive protein; OR, odds ratio; Q, quartile; RBP4, retinol-binding protein 4; SGA/LGA, small/large for gestational age.

[a] Unadjusted.

[b] Adjusted for maternal age, parity, systolic and diastolic BP, gestational week, pregnancy complications, assisted reproduction and fetal sex.

[c] Adjusted for model 2 variables plus blood lipids, hsCRP, liver and kidney function.

eTable 4. Associations of serum RBP4 levels with fetal growth indicators and SGA/LGA among nulliparous participants (N=7,110)

	Birth length, cm		Birthweight, g		SGA		LGA	
	β (95% CI)	*P* value	β (95% CI)	*P* value	OR (95% CI)	*P* value	OR (95% CI)	*P* value
Model 1[a]								
Q1 (<28.8 mg/L)	Ref.		Ref.		Ref.		Ref.	
Q2 (28.8–34.0 mg/L)	-0.00 (-0.09 to 0.09)	1.0	-33.49 (-65.37 to -1.62)	0.04	1.33 (1.05–1.68)	0.019	0.60 (0.49–0.73)	<0.001
Q3 (34.1–40.1 mg/L)	0.14 (0.05–0.23)	0.003	-16.31 (-48.45 to 15.84)	0.3	1.50 (1.19–1.89)	<0.001	0.63 (0.52–0.77)	<0.001
Q4 (>40.1 mg/L)	0.07 (-0.02 to 0.16)	0.1	-44.72 (-76.79 to -12.65)	0.006	1.85 (1.48–2.32)	<0.001	0.58 (0.48–0.71)	<0.001
P for trend		0.027		0.020		<0.001		<0.001
Continuous	0.00 (-0.00 to 0.01)	0.060	-2.07 (-3.37 to -0.77)	0.002	1.03 (1.02–1.04)	<0.001	0.98 (0.97–0.98)	<0.001
Model 2[b]								
Q1 (<28.8 mg/L)	Ref.		Ref.		Ref.		Ref.	
Q2 (28.8–34.0 mg/L)	-0.08 (-0.15 to -0.01)	0.021	-60.07 (-84.10 to -36.04)	<0.001	1.37 (1.08–1.75)	0.011	0.56 (0.46–0.69)	<0.001
Q3 (34.1–40.1 mg/L)	-0.05 (-0.12 to 0.02)	0.2	-89.18 (-113.49 to -64.87)	<0.001	1.70 (1.34–2.16)	<0.001	0.55 (0.45–0.68)	<0.001
Q4 (>40.1 mg/L)	-0.11 (-0.18 to -0.04)	<0.001	-118.88 (-143.21 to -94.56)	<0.001	2.16 (1.71–2.73)	<0.001	0.50 (0.40–0.61)	<0.001
P for trend		0.006		<0.001		<0.001		<0.001
Continuous	-0.00 (-0.01 to -0.00)	0.003	-5.38 (-6.37 to -4.40)	<0.001	1.03 (1.03–1.04)	<0.001	0.97 (0.96–0.98)	<0.001
Model 3[c]								
Q1 (<28.8 mg/L)	Ref.		Ref.		Ref.		Ref.	

Q2 (28.8–34.0 mg/L)	-0.08 (-0.15 to -0.01)	0.020	-56.15 (-80.31 to -31.98)	<0.001	1.34 (1.04–1.73)	0.025	0.57 (0.46–0.71)	<0.001
Q3 (34.1–40.1 mg/L)	-0.05 (-0.12 to 0.03)	0.2	-80.01 (-104.94 to -55.07)	<0.001	1.60 (1.24–2.07)	<0.001	0.59 (0.47–0.73)	<0.001
Q4 (>40.1 mg/L)	-0.11 (-0.19 to -0.04)	0.003	-108.75 (-134.70 to -82.80)	<0.001	2.02 (1.55–2.62)	<0.001	0.53 (0.42–0.67)	<0.001
P for trend		0.011		<0.001		<0.001		<0.001
Continuous	-0.00 (-0.01 to -0.00)	0.003	-5.21 (-6.28 to -4.14)	<0.001	1.03 (1.02–1.05)	<0.001	0.97 (0.96–0.98)	<0.001

BMI, body mass index; BP, blood pressure; CI, confidence interval; hsCRP, high sensitive C-reactive protein; OR, odds ratio; Q, quartile; RBP4, retinol-binding protein 4; SGA/LGA, small/large for gestational age.

[a] Unadjusted.

[b] Adjusted for maternal age, BMI, systolic and diastolic BP, gestational week, pregnancy complications, assisted reproduction and fetal sex.

[c] Adjusted for model 2 variables plus blood lipids, hsCRP, liver and kidney function.

eTable 5. Associations of serum RBP4 levels with fetal growth indicators and SGA/LGA among participants with natural fertility (N=11,575)

	Birth length, cm		Birthweight, g		SGA		LGA	
	β (95% CI)	*P* value	β (95% CI)	*P* value	OR (95% CI)	*P* value	OR (95% CI)	*P* value
Model 1[a]								
Q1 (<28.8 mg/L)	Ref.		Ref.		Ref.		Ref.	
Q2 (28.8–34.0 mg/L)	0.05 (-0.02 to 0.13)	0.2	-20.34 (-46.00 to 5.33)	0.1	1.29 (1.05–1.57)	0.013	0.70 (0.61–0.80)	<0.001
Q3 (34.1–40.1 mg/L)	0.12 (0.05–0.19)	0.002	-27.21 (-52.85 to -1.57)	0.038	1.47 (1.21–1.78)	0.002	0.66 (0.58–0.76)	<0.001
Q4 (>40.1 mg/L)	0.04 (-0.03 to 0.12)	0.3	-60.17 (-85.83 to -34.51)	<0.001	1.95 (1.62–2.34)	<0.001	0.57 (0.49–0.66)	<0.001
P for trend		0.2		<0.001		<0.001		<0.001
Continuous	0.00 (-0.00 to 0.01)	0.1	-2.58 (-3.61 to -1.55)	<0.001	1.03 (1.02–1.04)	<0.001	0.98 (0.97–0.98)	<0.001
Model 2[b]								
Q1 (<28.8 mg/L)	Ref.		Ref.		Ref.		Ref.	
Q2 (28.8–34.0 mg/L)	-0.03 (-0.09 to 0.02)	0.2	-52.25 (-71.63 to -32.86)	<0.001	1.32 (1.07–1.62)	0.008	0.67 (0.58–0.77)	<0.001
Q3 (34.1–40.1 mg/L)	-0.06 (-0.11 to -0.00)	0.046	-94.59 (-114.01 to -75.16)	<0.001	1.64 (1.34–2.01)	<0.001	0.59 (0.51–0.69)	<0.001
Q4 (>40.1 mg/L)	-0.13 (-0.19 to -0.07)	<0.001	-131.28 (-150.80 to -111.76)	<0.001	2.19 (1.80–2.66)	<0.001	0.50 (0.43–0.58)	<0.001
P for trend		<0.001		<0.001		<0.001		<0.001
Continuous	-0.01 (-0.01 to -0.00)	<0.001	-5.80 (-6.58 to -5.02)	<0.001	1.03 (1.03–1.04)	<0.001	0.97 (0.96–0.98)	<0.001
Model 3[c]								
Q1 (<28.8 mg/L)	Ref.		Ref.		Ref.		Ref.	

Q2 (28.8–34.0 mg/L)	-0.05 (-0.11 to 0.01)	0.074	-52.09 (-71.51 to -32.66)	<0.001	1.32 (1.07–1.64)	0.011	0.68 (0.58–0.79)	<0.001
Q3 (34.1–40.1 mg/L)	-0.07 (-0.13 to -0.01)	0.015	-87.62 (-107.49 to -67.75)	<0.001	1.60 (1.29–1.99)	<0.001	0.63 (0.54–0.74)	<0.001
Q4 (>40.1 mg/L)	-0.15 (-0.21 to -0.09)	<0.001	-124.74 (-145.45 to -104.02)	<0.001	2.15 (1.73–2.67)	<0.001	0.52 (0.44–0.62)	<0.001
P for trend		<0.001		<0.001		<0.001		<0.001
Continuous	-0.01 (-0.01 to -0.00)	<0.001	-5.71 (-6.56 to -4.87)	<0.001	1.03 (1.03–1.04)	<0.001	0.97 (0.96–0.98)	<0.001

BMI, body mass index; BP, blood pressure; CI, confidence interval; hsCRP, high sensitive C-reactive protein; OR, odds ratio; Q, quartile; RBP4, retinol-binding protein 4; SGA/LGA, small/large for gestational age.

[a] Unadjusted.

[b] Adjusted for maternal age, BMI, parity, systolic and diastolic BP, gestational week, pregnancy complications, and fetal sex.

[c] Adjusted for model 2 variables plus blood lipids, hsCRP, liver and kidney function.

eTable 6. Associations of serum RBP4 levels with fetal growth indicators and SGA/LGA among participants without pregnancy complications (N=9,679)

	Birth length, cm		Birthweight, g		SGA		LGA	
	β (95% CI)	*P* value	β (95% CI)	*P* value	OR (95% CI)	*P* value	OR (95% CI)	*P* value
Model 1[a]								
Q1 (<28.8 mg/L)	Ref.		Ref.		Ref.		Ref.	
Q2 (28.8–34.0 mg/L)	0.07 (-0.00 to 0.14)	0.051	-14.84 (-41.65 to 11.97)	0.3	1.31 (1.05–1.63)	0.017	0.70 (0.60–0.82)	<0.001
Q3 (34.1–40.1 mg/L)	0.14 (0.06–0.21)	0.002	-15.93 (-42.60 to 10.75)	0.2	1.45 (1.16–1.80)	0.001	0.64 (0.55–0.75)	<0.001
Q4 (>40.1 mg/L)	0.13 (0.06–0.20)	0.003	-32.45 (-59.09 to -5.81)	0.017	1.78 (1.44–2.19)	<0.001	0.63 (0.54–0.74)	<0.001
P for trend		<0.001		0.020		<0.001		<0.001
Continuous	0.01 (0.00–0.01)	<0.001	-1.55 (-2.64 to -0.47)	0.005	1.02 (1.01–1.03)	<0.001	0.98 (0.97–0.99)	<0.001
Model 2[b]								
Q1 (<28.8 mg/L)	Ref.		Ref.		Ref.		Ref.	
Q2 (28.8–34.0 mg/L)	-0.03 (-0.08 to 0.03)	0.3	-53.27 (-74.02 to -32.52)	<0.001	1.33 (1.06–1.67)	0.015	0.67 (0.57–0.79)	<0.001
Q3 (34.1–40.1 mg/L)	-0.04 (-0.09 to 0.02)	0.1	-85.27 (-105.97 to -64.58)	<0.001	1.57 (1.25–1.96)	0.001	0.58 (0.49–0.69)	<0.001
Q4 (>40.1 mg/L)	-0.07 (-0.12 to -0.01)	0.017	-117.81 (-138.59 to -97.03)	<0.001	2.04 (1.64–2.54)	<0.001	0.54 (0.45–0.63)	<0.001
P for trend		0.016		<0.001		<0.001		<0.001
Continuous	-0.00 (-0.00 to -0.00)	0.017	-5.25 (-6.09 to -4.40)	<0.001	1.03 (1.02–1.04)	<0.001	0.97 (0.97–0.98)	<0.001
Model 3[c]								

Q1 (<28.8 mg/L)	Ref.		Ref.		Ref.		Ref.	
Q2 (28.8–34.0 mg/L)	-0.05 (-0.10 to 0.01)	0.089	-52.42 (-73.21 to -31.64)	<0.001	1.32 (1.04–1.67)	0.023	0.68 (0.57–0.80)	<0.001
Q3 (34.1–40.1 mg/L)	-0.06 (-0.11 to -0.00)	0.049	-78.19 (-99.36 to -57.02)	<0.001	1.53 (1.21–1.94)	<0.001	0.62 (0.52–0.75)	<0.001
Q4 (>40.1 mg/L)	-0.10 (-0.16 to -0.04)	0.002	-108.90 (-131.04 to -86.75)	<0.001	1.96 (1.54–2.50)	<0.001	0.57 (0.48–0.69)	<0.001
P for trend		0.002		<0.001		<0.001		<0.001
Continuous	-0.00 (-0.01 to -0.00)	0.001	-5.06 (-5.97 to -4.14)	<0.001	1.03 (1.02–1.04)	<0.001	0.97 (0.97–0.98)	<0.001

BMI, body mass index; BP, blood pressure; CI, confidence interval; hsCRP, high sensitive C-reactive protein; OR, odds ratio; Q, quartile; RBP4, retinol-binding protein 4; SGA/LGA, small/large for gestational age.

[a] Unadjusted.

[b] Adjusted for maternal age, BMI, parity, systolic and diastolic BP, gestational week, assisted reproduction, and fetal sex.

[c] Adjusted for model 2 variables plus blood lipids, hsCRP, liver and kidney function.

eTable 7. Associations of serum RBP4 levels with fetal growth indicators and SGA/LGA among participants without preterm birth (N=11,007)

	Birth length, cm		Birthweight, g		SGA		LGA	
	β (95% CI)	*P* value	β (95% CI)	*P* value	OR (95% CI)	*P* value	OR (95% CI)	*P* value
Model 1[a]								
Q1 (<28.8 mg/L)	Ref.		Ref.		Ref.		Ref.	
Q2 (28.8–34.0 mg/L)	-0.03 (-0.07 to 0.00)	0.085	-52.24 (-74.53 to -29.95)	<0.001	1.29 (1.04–1.60)	0.019	0.70 (0.61–0.81)	<0.001
Q3 (34.1–40.1 mg/L)	-0.04 (-0.08 to -0.00)	0.034	-75.71 (-97.93 to -53.49)	<0.001	1.53 (1.24–1.88)	<0.001	0.66 (0.57–0.76)	<0.001
Q4 (>40.1 mg/L)	-0.07 (-0.10 to -0.03)	<0.001	-106.44 (-128.58 to -84.30)	<0.001	1.91 (1.56–2.33)	<0.001	0.59 (0.51–0.68)	<0.001
P for trend		<0.001				<0.001		<0.001
Continuous	-0.00 (-0.01 to -0.00)	<0.001	-4.79 (-5.68 to -3.91)	<0.001	1.03 (1.02–1.04)	<0.001	0.98 (0.97–0.98)	<0.001
Model 2[b]								
Q1 (<28.8 mg/L)	Ref.		Ref.		Ref.		Ref.	
Q2 (28.8–34.0 mg/L)	-0.04 (-0.08 to -0.01)	0.015	-57.70 (-77.45 to -37.95)	<0.001	1.33 (1.07–1.66)	0.012	0.66 (0.57–0.76)	0.035
Q3 (34.1–40.1 mg/L)	-0.07 (-0.10 to -0.03)	<0.001	-97.54 (-117.28 to -77.80)	<0.001	1.68 (1.36–2.09)	<0.001	0.58 (0.50–0.68)	<0.001
Q4 (>40.1 mg/L)	-0.09 (-0.13 to -0.06)	<0.001	-134.10 (-153.84 to -114.36)	<0.001	2.17 (1.76–2.67)	<0.001	0.50 (0.43–0.59)	<0.001
P for trend		<0.001		<0.001		<0.001		<0.001
Continuous	-0.00 (-0.01 to -0.00)	<0.001	-6.02 (-6.81 to -5.22)	<0.001	1.03 (1.03–1.04)	<0.001	0.97 (0.96–0.98)	<0.001

Model 3[c]								
Q1 (<28.8 mg/L)	Ref.		Ref.		Ref.		Ref.	
Q2 (28.8–34.0 mg/L)	-0.04 (-0.07 to -0.00)	0.039	-53.84 (-73.54 to -34.14)	0.032	1.30 (1.04–1.64)	0.024	0.68 (0.58–0.79)	<0.001
Q3 (34.1–40.1 mg/L)	-0.05 (-0.09 to -0.02)	0.006	-86.66 (-106.75 to -66.56)	0.010	1.60 (1.27–2.01)	<0.001	0.63 (0.53–0.74)	<0.001
Q4 (>40.1 mg/L)	-0.08 (-0.12 to -0.04)	<0.001	-120.86 (-141.73 to -99.98)	<0.001	2.04 (1.62–2.56)	<0.001	0.54 (0.46–0.65)	<0.001
P for trend		<0.001		<0.001		<0.001		<0.001
Continuous	-0.00 (-0.01 to -0.00)	<0.001	-5.64 (-6.49 to -4.78)	<0.001	1.03 (1.02–1.04)	<0.001	0.97 (0.97–0.98)	<0.001

BMI, body mass index; BP, blood pressure; CI, confidence interval; hsCRP, high sensitive C-reactive protein; OR, odds ratio; Q, quartile; RBP4, retinol-binding protein 4; SGA/LGA, small/large for gestational age.

[a] Unadjusted.

[b] Adjusted for maternal age, BMI, parity, systolic and diastolic BP, gestational week, pregnancy complications, assisted reproduction, and fetal sex.

[c] Adjusted for model 2 variables plus blood lipids, hsCRP, liver and kidney function.
